# Pulsar glitches from quantum vortex networks

**DOI:** 10.1038/s41598-024-56383-w

**Published:** 2024-04-03

**Authors:** Giacomo Marmorini, Shigehiro Yasui, Muneto Nitta

**Affiliations:** 1 Department of Physics, Nihon University, Tokyo, Japan; 2 Department of Physics and Mathematics, Aoyama Gakuin University, Sagamihara, Kanagawa Japan; 3 International Institute for Sustainability with Knotted Chiral Meta Matter (SKCM2), Hiroshima University, Hiroshima, 739-8511 Japan; 4 Department of Physics and Research and Education Center for Natural Sciences, Keio University, Kanagawa, 223-8521 Japan

**Keywords:** Compact astrophysical objects, Nuclear astrophysics

## Abstract

Neutron stars or pulsars are very rapidly rotating compact stars with extremely high density. One of the unsolved long-standing problems of these enigmatic celestial bodies is the origin of pulsars’ glitches, i.e., the sudden rapid deceleration in the rotation speed of neutron stars. Although many glitch events have been reported, there is no consensus on the microscopic mechanism responsible for them. One of the important characterizations of the glitches is the scaling law $P(E) \sim E^{-\alpha }$ of the probability distribution for a glitch with energy *E*. Here, we reanalyse the accumulated up-to-date observation data to obtain the exponent $\alpha \approx 0.88$ for the scaling law, and propose a simple microscopic model that naturally deduces this scaling law without any free parameters. Our model explains the appearance of these glitches in terms of the presence of *quantum vortex networks* arising at the interface of two different kinds of superfluids in the core of neutron stars; a *p*-wave neutron superfluid in the inner core which interfaces with the *s*-wave neutron superfluid in the outer core, where each integer vortex in the *s*-wave superfluid connects to two half-quantized vortices in the *p*-wave superfluid through structures called “boojums”.

## Introduction

Neutron stars (NSs) and in particular pulsars are compact stars with the highest known density in our universe (about one solar mass within $10^3$ km$^3$)^1^, thereby providing an astrophysical laboratory to study phases of matter under extraordinary conditions: not only at very high density but also under rapid rotation and extremely strong magnetic fields (see Refs.^2,3^ for recent reviews). The study of NSs attracts great interest from researchers in diverse fields as recently there has been observations of highly massive NSs^4,5^ and gravitational waves from a binary NS merger^6^. One of the most intensive events of NSs is pulsar’s glitches, i.e., the abrupt deceleration of the rotation speed of NSs^7^ (see Refs.^8–10^ as recent reviews). Although many glitch events have been reported, the microscopic mechanism responsible for all the glitch events is still elusive. However, several ideas have been theoretically proposed: starquakes^11,12^ and the catastrophic unpinning of quantum superfluid vortices^13,14^. While star quakes are not a viable explanation for all glitches, vortex motion is generally invoked^15^.

One of the important characterizations of the glitches is the scaling law of the cumulative probability distribution of glitch sizes $P(E) \sim E^{-\alpha }$ with an exponent $\alpha$, giving the probability of the occurrence of a glitch with energy *E*^16^ (see also Ref.^17^). The value of $\alpha \approx 0.14$ was obtained in Ref.^16^ (we notice that the size distribution of glitches is not precisely a power law but bi-modal (multi-modal) with an excess of large glitches^9^). Adopting the scaling law for many glitches in average seems to be natural if one regards the glitches occurrence as consequences of starquakes in the crust region at the surface of NSs. In fact, this scaling law resembles the Gutenberg–Richter law expressing the probability distribution of the total number of earthquakes of certain magnitude^18^. This is known as an instance of power-law distributions commonly found in diverse subjects in the natural, human and social sciences; for example the Pareto distribution drawn from economics^19^ and scale-free networks such as the World Wide Web in network science^20^. The “network” is one of the key ingredients in our study.

**Figure 1:**
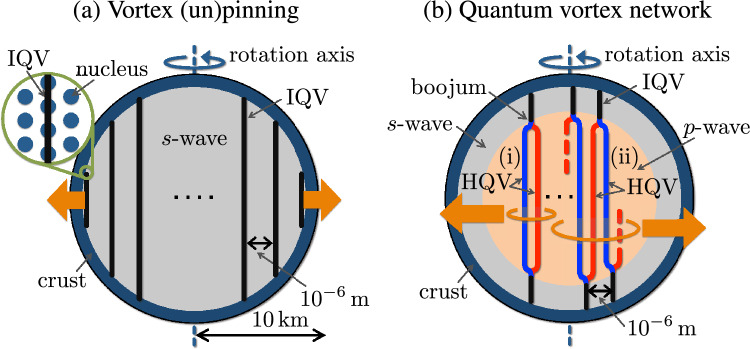
Inner structures of NSs. (**a**) The conventioanl picture: Pinned vortices in the *s*-wave superfluid in the core surrounded by the crust (dark blue) in the outermost region. Vortices form a lattice and are pinned at nuclei in the crust (see the inset). The orange arrows denote that vortices are released when a NS decreases its rotation speed. (**b**) Our picture: vortex network in the *p*-wave inner core (pink) surrounded by the *s*-wave outer core of the spherical shell (grey). Vortices are *not* pinned at nuclei in the crust. A single integer quantum vortex (IQV) in the *s*-wave outer core is split into two half-quantized vortices (HQVs) in the *p*-wave inner core. The red and blue vortices indicate different topological charges of the HQVs (±), respectively. There are two cases (i) and (ii) for the connections between IQVs and HQVs. (i) The same pair of two HQVs in the *p*-wave inner core are connected to a single IQV in the upper and lower *s*-wave regions, forming a cluster of a certain minimum size. (ii) Two HQVs connected to a single vortex in the upper (lower) *s*-wave region are connected to different IQVs in the lower (upper) *s*-wave region, forming a cluster with a larger size. In our proposal, this network in (ii) plays an essential role (see the text).

**Figure 2:**
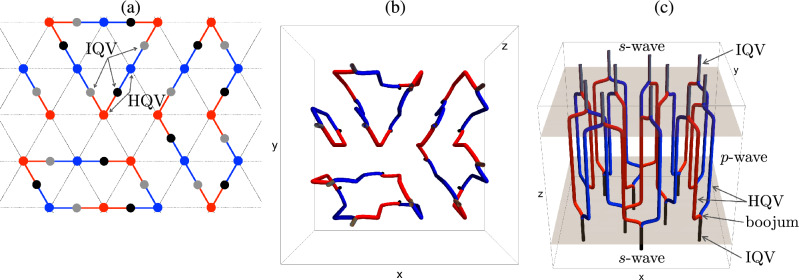
Schematic views of pairings of vortices at *s*-*p*-*s* interfaces resulting in vortex clusters. (**a**) Top view of a vortex network, (**b**) top view and (**c**) view from diagonally above of a 3D configuration. In (**a**), black and grey dots denote integer vortices in the *s*-wave region at the top and bottom, respectively, while red and blue dots and lines denote HQVs in the *p*-wave region forming an Abrikosov lattice. Clusters with sizes one, three and four are shown.

The other key ingredient of our study is *quantum vortices* in superfluids. Superfluids are fluids possessing zero viscosity which can support states with persistent flows. In laboratory experiments, $^4$He atoms become a superfluid at low temperatures. In the interiors of NSs, neutrons form Cooper pairs and exhibit superfluidity^21^ (see Ref.^22^ for a recent review) (see Fig. 1a), which is consistent with the thermal evolution of NSs, the long relaxation time after each glitch^11,12,23^, and the neutrino emissivity^24^. When superfluids undergo rotation, vortices are created. One of remarkable properties of superfluids is the fact that these vortices carry a quantized amount of angular momentum unlike classical fluids, hence the name *quantum vortices*. Since neutron stars are rapidly rotating, the superfluid component is pierced by quantum vortices lying along the rotation axis, which typically form an Abrikosov triangular lattice whose number can reach $10^{19}$. There is also an accompanying ordinary component (not forming Cooper pairs) which lowers its rotation speed by releasing gravitational waves and electromagnetic pulses, whose period is observed to be a continuous quantity. On the other hand, the superfluid component maintains a constant rotation speed, as a consequence of superfluidity. Therefore, a gap between the rotation speeds of the two components grows over time. The superfluid component can lower its rotation speed by releasing vortices. If the vortices can be released one by one, then the superfluid component’s rotation speed can catch up with the ordinary component *immediately* once this difference reaches the amount corresponding to one quantized vortex, and the change of the rotation should be (almost) smooth at any given time, which does not explain the appearance of glitches.

In order to overcome this problem, the hypothesis of catastrophic unpinning of quantum vortices^13,14^ assumes that all vortices are pinned to nuclei, which play the role of impurities in the crust (see the inset of Fig. 1a), analogously to metallic superconductors where Abrikosov vortices are energetically favored to be pinned to impurities. Then, in order to explain the occurrence of glitches, an avalanche of unpinning of a large number of vortices is assumed to occur spontaneously. Several models have been suggested, but one usually needs some phenomenological parameters in order to account for the momentum transfer from the core region to the crust, a subject around which there is quite some uncertainty, fueling a long debate^25–33^ (see Ref.^15^ as a review). Moreover, a recent work based on microscopic calculations^34^ shows that, unlike in metallic superconductors, pinning of vortices to impurities is energetically disfavored in the case of nuclear superfluids (see, e.g., Ref.^35^ for the recent study) (despite the advances in microscopic calculations, the relevance of the pinning to the glitch mechanism is still unclear because the sign of the single-nucleus interaction may not be directly related to the absence of the pinning^36^).

Here, we first reanalyse the accumulated up-to-date observation data^37,38^ to obtain the scaling law $P(E) \sim E^{-\alpha }$ with the exponent $\alpha \approx 0.88$. We then propose a simple microscopic model that naturally deduces the scaling law with this exponent without any additional free parameters. Our model explains the origin of the glitches in terms of a *quantum vortex network* that arises at the interfaces of the two different kinds of superfluids in the cores of NSs. In contrast to the catastrophic vortex unpinning hypothesis, we need no (un)pinning for the accumulation of quantum vortices.

The two different kinds of superfluids explained above were theoretically predicted as two different types of neutron Cooper pairs: *s*-wave and *p*-wave parings. While an *s*-wave paring^21^ is dominant in the low-density regime relevant for the neutron star outer core, a *p*-wave paring is dominant in the high-density regime relevant for the inner core^39,40,40–49^ (more precisely, *s*-wave and *p*-wave pairings denote $^1S_0$ (spin-singlet and *s*-wave with total angular momentum $J=0$) and ^3^*P*_2_ (a spin-triplet and *p*-wave with total angular momentum $J=2$) parings, respectively. See Supplementary Information for more details). Therefore, we will assume that the interior of neutron stars consists of a layer structure with a *p*-wave inner core surrounded by an *s*-wave outer core forming a spherical shell (Fig. 1b) (As a remark for a different argument, the the Kelvin–Helmholtz instability at the interface of the *s*-wave crust and the *p*-wave core may provide a trigger mechanism for pulsar glitches^50^. This is different from the mechanism proposed in our study). We will focus on the superfluid components neglecting interaction with a normal component, while the mutual friction between normal and superfluid components exists in real astrophysical systems^10,51^.

We show that the glitch mechanism can be explained by the crucial property of *p*-wave superfluids: the existence of *half-quantized* vortices (HQVs) carrying half-quantized circulations^52–55^, as opposed to integer-quantized vortices (IQVs)^44,46,47,56,57^. HQVs are energetically favored so that one IQV is split into two HQVs (denoted as red and blue vortices in Fig. 1b) with additional topological charges cancelling each other (see Supplementary Information)^53,54^. Here the colors of red and blue are used to distinguish the two species of topological charges of the HQVs (±). Quantum vortices in the *s*-wave and *p*-wave superfluids are connected through junctions called “boojums”^58^ at the interface; *one* IQV in the *s*-wave superfluid is connected to *two* HQVs in the *p*-wave superfluid. We thus have a picture of a large number of vortices penetrating the *p*-wave inner core surrounded by the *s*-wave outer core as in Fig. 1b. Then, it is possible that the same pair of two HQVs in the *p*-wave core are connected to a single IQV in the upper and lower *s*-wave regions (see (i) in Fig. 1b). If this is the case, a neutron star superfluid can change its rotation speed by releasing vortices one by one from the core as is the case without vortex pinning at the crust, which would not explain the appearance of glitches. However, more generally, as illustrated in (ii) in Fig. 1b, we can have two HQVs connected to a single IQV in the upper (lower) *s*-wave region which are connected to a different IQV in the lower (upper) *s*-wave region, thereby leading to the formation of a vortex network composed of a cluster of connected vortices as in Fig. 2. As we show below, in this case, each cluster can contain a large number of vortices, which exhibits the power-law distribution of glitches.

**Figure 3:**
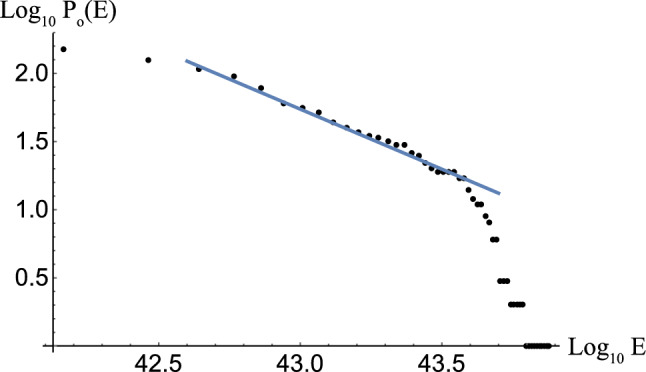
$\log _{10} P_{o}(E)$ v.s. $\log _{10} E$ plot of observed glitches of energy *E*. The blue line indicates Eq. (1) obtained by fit using all the data points, for which the intercept is arbitrary for the log-log plots.

### Observed distribution of energy

Let us analyze the glitch dataset reported by Ref.^37^; we additionally use the pulsar catalogue^38^ to retrieve the pulsar periods. Let $P_o(E)$ denote the cumulative probability of observed glitch energies as in Ref.^16^ (see “Methods” for details). Here the glitch energy *E* denotes the released kinetic energy $I\Omega \Delta \Omega$ with the momentum of inertia *I* of the NS, the rotation frequency $\Omega$ of the NS, and the change of frequency $\Delta \Omega$ by the glitch. Figure 3 displays the log-log plot of $P_o(E)$ obtained from the analysis of 533 glitches, where the line, determined by the least squares method using all 533 data points, shows the cumulative distribution of the power law

1 $$\begin{aligned} P_o(E)\sim E^{-0.88\pm 0.03}. \end{aligned}$$

One can see that in the central region (away from extremely small or extremely large glitches), the cumulative distribution is well approximated by the power law. However, note that we used all the data without any cutoff for fitting.

### Cluster size and energy distribution

The radius of the whole core is typically 10 km. There is an uncertainty for the size of the *p*-wave inner core but it is thought to be a few km. The mean intervortex distance is of order $10^{-6}$ m, and the number of vortices is of order $10^{19}$. We assume that the *s*–*p* interfaces are mostly flat and parallel for simplicity although the outer and inner cores would be almost spherical. However, our results do not depend on the precise details of the underlying shapes and sizes at all. We further assume a triangular vortex lattice which is rigidly rotating, as is typically the case for superfluids. We consider a rotating frame in which this lattice is static. Then, at the *s*–*p* interface, two HQVs with different topological charges in the *p*-wave region will pair and connect to an integer vortex in the *s*-wave region via a boojum.

**Figure 4:**
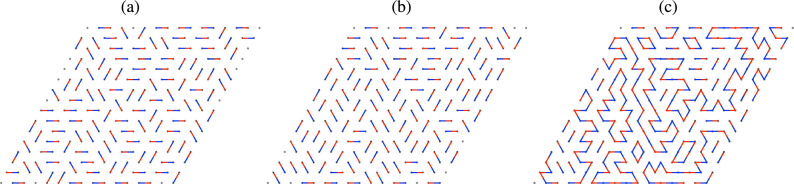
Snapshot of pairings at the top (**a**) and bottom (**b**) interface, and the resulting vortex clusters (**c**), which appears as loops in the top view. Here, the red and blue dots and segments denote HQVs in the *p*-wave region.

In order to study the cluster size distribution we employ the following model. We assume that the HQVs in the *p*-wave region form an Abrikosov-like triangular lattice with periodic boundary conditions; however we do not assume any restriction on the blue/red vortex pattern except that for a red vortex it is always possible to find a blue nearest neighbor and vice versa. We then consider a triangular vortex lattice of rhombic shape and simulate two random blue-red pairings, that would correspond to the boojums at the top and bottom ends of the vortices. The superposition of the two pairings generates loops of various sizes, which corresponds to clusters in which all vortices are connected via boojums. This was schematically depicted in Fig. 2, and an example of configurations taken from our simulation is presented in Fig. 4 (note that a minimal loop made of two vortices appears in the form of a segment).

**Figure 5:**
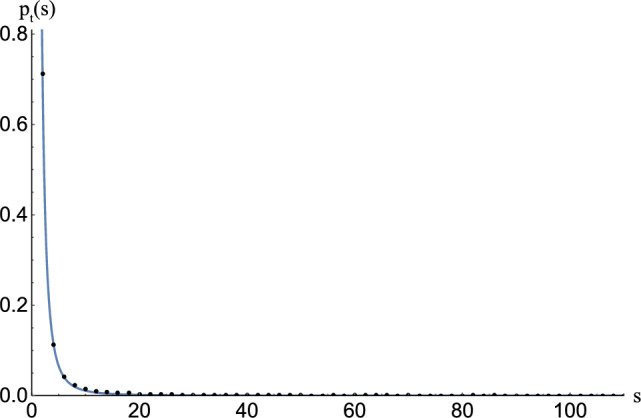
Plot in linear scale of the simulated cluster-size probability data points $\{s_i,q_i=p(s_i)\}$ and the inferred power law $p_t(s) \sim s^{-2.6 }$ (see “Methods” for details).

Statistical analysis of the cluster size distribution shows a power-law behaviour (for details of the analysis see “Methods”). The best estimate of the probability distribution of cluster size is

2 $$\begin{aligned} p_t(s) \sim s^{-2.6\pm 0.3}, \end{aligned}$$

where *s* is the cluster size and the subscript *t* stands for “theoretical”; the simulated data points and the best fit to Eq. (2) is displayed in Fig. 5. Then, we can define the cumulative probability as $P_t(s) = \int _s^{s_{max}} p_t(u) du$. A cluster of size *s* defines a region inside of which the number of vortices is of order $s^2$. Since there is no reconnection between HQVs with different topological charges^59,60^, when a cluster is expelled from the neutron star core, it necessarily drags all the other vortices enclosed by that cluster. It is therefore safe to assume that the energy associated with the emission of a vortex cluster satisfies the relation $E=c s^2$ for some constant *c*. By using this relation, we can translate the size distribution in Eq. (2) to the energy probability distribution, $p_t(E) \sim E^{-1.8\pm 0.2}$ and the corresponding cumulative distribution (see “Methods”)

3 $$\begin{aligned} P_t(E) \sim E^{-0.8\pm 0.2}. \end{aligned}$$

Thus, our model gives a description of the scaling law in Eq. (1) for the set of observed glitches without any free parameters.

### Conclusion

We have obtained the power-law scaling law of Eq. (1) for glitches from recent observational data, and proposed a simple model to explain the scaling law of Eq. (3) based on vortices penetrating the *p*-wave superfluid core surrounded by the *s*-wave superfluid. Boojums connecting two HQVs in the *p*-wave superfluid and one integer vortex in the *s*-wave superfluid give rise to clusters of vortices, whose distributions realise the scaling law. The appearance of the vortex network naturally explains a similarity with power-law distributions in other systems widely discussed in network science. The key strength of our approach is that our model contains no free parameters to explain the observed data.

Explaining deviations from the power laws such as excesses of large events, see e.g. Ref.^51^, is a future problem. Future models should also incorporate the hydrodynamics on the neutron star fluids, which can impact significantly also the observed size distributions and may explain the deviations from the power laws.

## Methods

### Observed distribution of glitch energy

Let us analyze the glitch dataset reported by Ref.^37^; we also use the pulsar catalogue^38^ to retrieve the pulsar periods. The energy of a glitch can be estimated from the change in rotational energy of the neutron star, namely $E_g= I\,\Omega \, \Delta \Omega$, where *I* is the moment of inertia and $\Omega$ is the rotational frequency. By taking $1.4M_\odot$ as the typical neutron star mass and $10^5$ cm as the typical radius, the glitch energy becomes^16^

4 $$\begin{aligned} E_g \approx \frac{a\cdot 10^{46}}{P^2} \frac{\Delta \nu }{\nu } \textrm{ergs}, \end{aligned}$$

where *P* is the period of the pulsar in seconds, $\Delta \nu / \nu$ is the relative change of frequency associated with the glitch and *a* is a constant of order 1 (for concreteness we take $a=2.91$^16^). Let us also define the cumulative probability of observed glitch energies as in Ref.^16^:

5 $$\begin{aligned} P_o(E) = \int _E^{E_{max}} p_o(u) du. \end{aligned}$$

Figure 3 displays the log-log plot of $P_o(E)$ obtained from the analysis of 533 glitches, where the line, determined by the least squares method using all 533 data points, shows the cumulative distribution of the power law in Eq. (1). Note that a change of the overall energy scale in Eq. (4) would not affect this behaviour.

This scaling law is significantly different from the previously found scaling behaviour $P_o(E) \sim E^{-0.14}$^16^. The reason can be considered as follows: Ref.^16^ used 27 data points of glitches in the energy range $10^{39}$–$10^{42}$ erg, for which the upper limit came from the observational limitations at that time, while our analysis with 533 data sets covers a broader range of energies than theirs per Fig. 3, which yields the observed differences of the exponents. Another important point is that several datum corresponding to extremely giant glitches with energy higher than $10^{43.6}$ erg are rare events, with corresponding lower probability, giving a smaller contribution to our fitting.

### Simulation of vortex pairing and clustering

**Figure 6:**
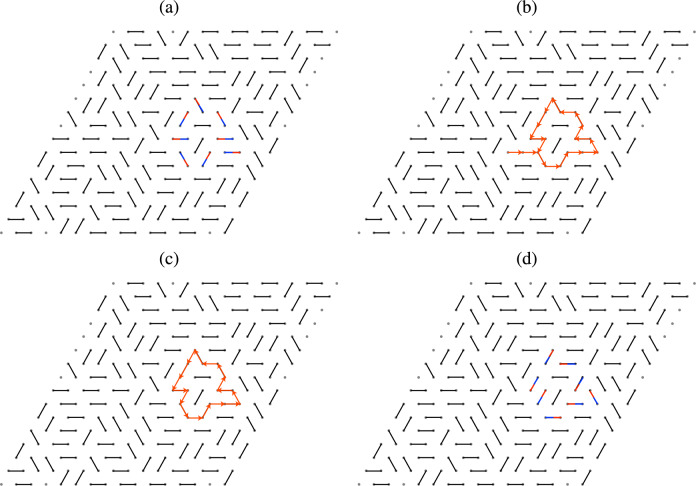
Schematic representation of the worm algorithm: (**a**) starting configuration; (**b**) random generation of the worm, which terminates when it crosses itself; (**c**) only the loop section of the worm is retained; (**d**) pairs are flipped along the worm, giving rise to the new configuration. In (**a**,**d**), only pairs before and after flipping are coloured by red and blue.

Cluster configurations are generated by simulating two uncorrelated random pairings in a triangular lattice with periodic boundary conditions. This is achieved by a version of the worm algorithm. First, we choose an arbitrary pairing, e.g. the simple pairing in Fig. 6, we then perform a series of decorrelation steps to obtain random pairings. Each step consists of the following operations. First we generate a path defined by a sequence of sites as follows: we randomly choose a site, that will be the first in the sequence; then we add the site to which the first one is paired; next, we randomly choose one of the five available nearest neighbours of the latter site (if it is a paired site already, which is already in the sequence, then it cannot be chosen) and add it to the sequence, followed by the corresponding paired site, and so on, until we encounter a site that is already in the sequence. This defines a path that contains a loop. If the loop has an odd number of segments, we discard it; otherwise we flip the pairing along the loop. This completes one decorrelation step; we perform $10^3$ steps for each site to obtain the first configuration (interpreted as the boojums at the top *s*–*p*-wave interface) and the same number of steps to obtain the second configuration (interpreted as the boojums at the bottom *s*–*p* interface). Superposing the two configurations gives a schematic top view of the HQV system, in which loops represent vortex clusters that are kept together by boojums. The size of the clusters is calculated by a standard analysis of the adjacency matrix, which is the accepted procedure for complex networks. See Supplementary Information (Sec. 3) for possible realisations in condensed matter setups of *s*–*p*–*s* interfaces.

### Statistical analysis

In this section, we statistically analyze 100 independent simulations of size with $48^2=2304$ sites. We use a Bayesian technique based on Dirichlet distributions^61^, which is appropriate for discrete random variables, such as our cluster size. The likelihood of observing a data set is given by the multinomial distribution

6 $$\begin{aligned} p(n_1,\ldots , n_k|q_1,\ldots ,q_k) = \frac{N!}{n_1!\ldots n_k!} \prod _i^k q_i^{n_i}, \end{aligned}$$

where $n_i$ is the number of observed clusters with size $s_i=2 i$, $N=\sum _i n_i$ and $q_i$ are the corresponding probabilities. Since our simulations are independent, we simply consider the data from all simulations at the same time. We can infer the the probabilities $\textbf{q}=\{q_i\}$ given the observed counts $\textbf{n}=\{n_i\}$ using Bayes’ theorem,

7 $$\begin{aligned} p(\textbf{q} | \textbf{n}) = \frac{p(\textbf{n} | \textbf{q}) \, p(\textbf{q}) }{\displaystyle {\int } d\textbf{q}\, p(\textbf{n} | \textbf{q}) \, p(\textbf{q}) }, \end{aligned}$$

where $p(\textbf{n} | \textbf{q})$ is the likelihood defined in Eq. (6) and $p(\textbf{q})$ is the prior distribution of the probabilities $\textbf{q}$. The appropriate choice of prior is the Dirichlet distribution

8 $$\begin{aligned} \textrm{Dir}(\textbf{q} | \textbf{a} ) = \frac{\Gamma (A)}{\prod _i^k \Gamma (a_i) } \prod _i^k q_i^{a_i}, \qquad A\equiv \sum _i^k a_i \end{aligned}$$

with $a_i=1, \, \forall i$; this is chosen since we do not assume any specific knowledge about the probabilities before doing the simulations. For the properties of the Dirichlet and multinomial distribution the posterior distribution will also be of Dirichlet type, namely

9 $$\begin{aligned} p(\textbf{q} | \textbf{n}) = \textrm{Dir}(\textbf{q} | \textbf{a} + \textbf{n} ). \end{aligned}$$

The expectation value of the probabilities will then be

10 $$\begin{aligned} \bar{q}_i = \frac{n_i+1}{N+k}. \end{aligned}$$

**Figure 7:**
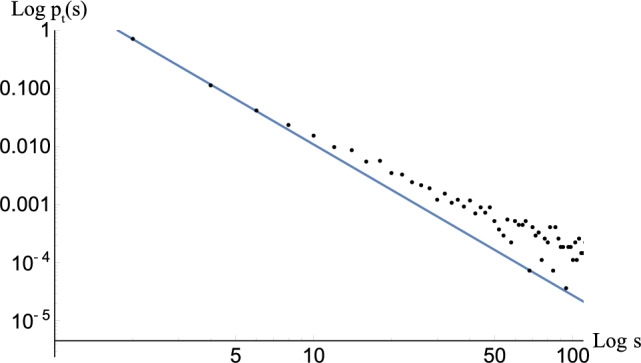
Plot in logarithmic scale of the simulated cluster-size probability data points $\{s_i,q_i=p(s_i)\}$ and the inferred power law $p_t(s) \sim s^{-2.6 }$.

The error bars for the $q_i$’s can be estimated by taking the square root of the variance $\textrm{Var}[q_i]= \textrm{E}[(q_i-\bar{q}_i)^2]$ over the distribution Eq. (9); they turn out to be quite small, essentially invisible in Fig. 7. We try to fit the data $\{s_i,q_i\}$ with two different models, namely exponential and power law, with a least-squares method, in order to understand the general behaviour of the cluster size distribution. By looking at the Akaike information criterion (see Supplementary Information (Sec. 4) for details), it is clear that that the power-law model is preferred and the best fit gives

11 $$\begin{aligned} p(s) \sim s^{-2.6 \pm 0.3} \end{aligned}$$

where $\pm 0.3$ denotes the statistical error. In Fig. 5 in the main text and in Fig. 7 the data $\{s_i,q_i\}$ and the inferred law of Eq. (11) are represented both in linear and logarithmic scales, respectively. Figure 7 shows the log-log plot, in which the line is determined by the least square method using all 100 simulations. The large size configurations on the right part deviate from the line, but these deviations are quite tiny ($\approx 10^{-4}$ to $10^{-3}$) giving a smaller contribution to our fitting. In fact, one can find that the fitting is very good in the linear plot.

We have also generated simulations with smaller *N* and get similar results, thereby implying the *N* independence of our results for large *N*.

### Translation from the cluster size distribution to the glitch energy distribution

By using the relation $E= c s^2$ between the glitch energy *E* and the vortex cluster size *s*, we can translate the size distribution in Eq. (11) to the (cumurative) energy distribution of glitchs as

12 $$\begin{aligned} P_t(s)= & {} 4.8\int _s^{s_{max}} u^{-2.6} du = c' \int _{E=cs^2}^{E_{max}=cs^2_{max}} v^{-1.3} \frac{dv}{v^{1/2}} \nonumber \\= & {} c' \int _{E}^{E_{max}} v^{-1.8} dv = c'' E^{-0.8} = P_t(E) \end{aligned}$$

with some constants $c'$ and $c''$, which defines the energy probability distribution, $p_t(E) = c' E^{-1.8\pm 0.2}$ and the corresponding cumulative distribution

13 $$\begin{aligned} P_t(E) = c'' E^{-0.8\pm 0.2}. \end{aligned}$$

### Supplementary Information

Supplementary Information.

## Data Availability

Our data are available upon request. The contact researcher is GM.

## Code availability

Our code is available upon request. The contact researcher is GM.
