# Supplementary material for: Pulsar glitches from quantum vortex networks
Source: Sci Rep. 2024 Apr 3;14:7857. doi: 10.1038/s41598-024-56383-w (PMC11322539; doi:10.1038/s41598-024-56383-w)
Supplement: Supplementary file 1 — Supplementary Information. [file 41598_2024_56383_MOESM1_ESM.pdf]

SUPPLEMENTARY INFORMATION FOR "PULSAR GLITCHES FROM QUANTUM VORTEX NETWORKS"

Giacomo Marmorini, Shigehiro Yasui and Muneto Nitta

1. *Comments on p-wave paring and half-quantized vortices*

As mentioned in the main text, in the interiors of NSs, neutrons form Cooper pairs and exhibit superfluidity [21] (see Refs. [22], [S1, S2] for recent reviews). Migdal considered an s-wave ($^1S_0$: spin-singlet and s-wave with total angular momentum $J = 0$) paring [21] as conventional (s-wave) metallic superconductors. Although the $^1S_0$ channel is attractive dominantly in the low-density regime, it becomes repulsive in the high-density regime in the neutron star core. There, the attraction is provided by the $^3P_2$ channel with the spin-orbit (LS) force inducing the neutron $^3P_2$ (spin-triplet and p-wave with total angular momentum $J = 2$) superfluids [39-49, 52-57], [S3–S20]. The ground state at the weak coupling limit is in the so-called nematic phase [45, 46]. We also comment that, although the boundary between the s- and p-wave regions may not be sharp microscopically (in reality an overlap region is predicted to exist [S20]), from a macroscopic point of view one can assume the presence of such an interface.

The p-wave pairings are widely considered in condensed matter physics such as p-wave superconductors for which electrons form Cooper pairs and ^{3}He superfluids for which ^{3}He atoms form Cooper pairs. Recently, p-wave superconductors (superfluids) have attracted great interests as topological superconductors (superfluids) [S21, S22], allowing topologically protected Majorana fermions on the boundary surfaces and vortex cores. In fact, the $^3P_2$ neutron superfluids have also been shown to be topological superfluids admitting surface Majorana fermions [S16] and Majorana bound states in their vortex cores [53, 57]. Thus, neutron star cores may be the largest topological materials in our universe.

The order parameter for p-wave paring (for $^3P_2$ neutron superfluids) is described by a 3×3 traceless symmetric matrix A with complex components [45, 46]. Then, vortices take the following forms: an IQV is represented as

$$A = e^{i\theta} A_0 \tag{S1}$$

with a spatial angle θ ($0 \le \theta < 2\pi$) around the vortex core [44, 46, 47, 56], where A_0 is a constant matrix representing the ground state configuration. On the other hand, a HQV takes the form of

$$A = e^{i\frac{\theta}{2}} O(\theta) A_0 O^T(\theta), \quad O(\theta) = \begin{pmatrix} \cos\frac{\theta}{4} & \pm\sin\frac{\theta}{4} & 0 \\ \mp\sin\frac{\theta}{4} & \cos\frac{\theta}{4} & 0 \\ 0 & 0 & 1 \end{pmatrix}, \tag{S2}$$

with the D_4 biaxial nematic ground state $A_0 \sim \mathrm{diag.}(1, -1, 0)$, where the sign $\pm$ ($\mp$) corresponds to HQVs with different topological charges cancelling each other [52], denoted by red and blue in the main text. The exponential factors $e^{i\theta}$ and $e^{i\frac{\theta}{2}}$ for IQV and HQV are the origin of integer and half quantizations, respectively. Importantly, the HQVs were recently observed in ^{3}He-A phase, as they were created with rotation or by the Kibble-Zurek mechanim and identified by the experimental technique on the nuclear magnetic resonance [S23].

2. *A story of the boojum*

Originally the boojum is a particular variety of the fictional animal species called *snarks* created by Lewis Carroll in his nonsense poem "The Hunting of the Snark." The similar structures found in helium superfluids were named boojums by Mermin [58]. See [S24] for the story how he made "boojum" an internationally accepted scientific term. Now boojums have been predicted to occur in ^{3}He superfluids [S25] in particular at the A-B phase boundary [S26, S27], liquid crystals [S28], Bose-Einstein condensates [S29], quantum field theory [S30–S32] and high density quark matter relevant for neutron star cores [S33–S35].

3. *Proposals for laboratory experiments*

We further point out that our mechanism for glitches by vortex networks through boojums can be simulated by laboratory experiments. One is with ultracold atomic gases, a mixture of a scalar Bose-Einstein condensate (BEC) and a spin-2 nematic BEC [S36–S38] (see Ref. [S39] for a review of spinor BEC), where the Gross-Pitaevskii equation for the latter is the same as the Ginzburg-Landau equation for a $^3P_2$ superfluid: a scalar BEC sandwiched between two spin-2 BECs. The other is ^{3}He superfluids with the A-B phase boundary on which one vortex in the A-phase is connected to two vortices in the B-phase through a boojum [S26], therefore an A-B-A phase configuration is relevant (a B-A-B phase configuration was experimentally realized [S27].

4. *Akaike Information Criterion (AIC)*

The Akaike Information Criterion (AIC) is defined as

$$\mathrm{AIC} = 2k - 2\ln(\hat{L}), \tag{S3}$$

where $\hat{L}$ is the maximum of the likelihood function L (often simply called the likelihood), which is formed from the joint probability distribution of a sample of data given a set of model parameter values; it is viewed and used as a function of the parameters given by the data sample. Generally, a lower AIC indicates a better fit.

[S1] N. Chamel. Superfluidity and superconductivity in neutron stars. *J. Astrophys. Astron.*, 2017.

[S2] B. Haskell and A. Sedrakian. Superfluidity and Superconductivity in Neutron Stars. *Astrophys. Space Sci. Libr.*, 457:401–454, 2018.

[S3] T. Fujita and T. Tsuneto. The ginzburg-landau equation for $^3P_2$ pairingsuperfluidity in neutron stars. *Prog. Theor. Phys.*, 48(3):766–782, 1972.

[S4] L. Amundsen and E. Ostgaard. Superfluidity of neutron matter (II). triplet pairing. *Nucl. Phys.*, A442:163–188, 1985.

[S5] T. Takatsuka and R. Tamagaki. Superfluidity in neutron star matter and symmetric nuclear matter. *Prog. Theor. Phys. Suppl.*, 112:27–66, 1993.

[S6] M. Baldo, J. Cugnon, A. Lejeune, and U. Lombardo. Proton and neutron superfluidity in neutron star matter. *Nucl. Phys.*, A536:349–365, 1992.

[S7] O. Elgaroy, L. Engvik, M. Hjorth-Jensen, and E. Osnes. Triplet pairing of neutrons in beta stable neutron star matter. *Nucl. Phys.*, A607:425–441, 1996.

[S8] V. A. Khodel, V. V. Khodel, and J. W. Clark. Universalities of triplet pairing in neutron matter. *Phys. Rev. Lett.*, 81:3828–3831, 1998.

[S9] M. Baldo, O. Elgaroey, L. Engvik, M. Hjorth-Jensen, and H. J. Schulze. Triplet $^3P_2$ to $^3F_2$ pairing in neutron matter with modern nucleon-nucleon potentials. *Phys. Rev.*, C58:1921–1928, 1998.

[S10] V. V. Khodel, V. A. Khodel, and J. W. Clark. Triplet pairing in neutron matter. *Nucl. Phys.*, A679:827–867, 2001.

[S11] M. V. Zverev, J. W. Clark, and V. A. Khodel. $^3P_2$-$^3F_2$ pairing in dense neutron matter: The Spectrum of solutions. *Nucl. Phys.*, A720:20–42, 2003.

[S12] S. Maurizio, J. W. Holt, and P. Finelli. Nuclear pairing from microscopic forces: singlet channels and higher-partial waves. *Phys. Rev.*, C90(4):044003, 2014.

[S13] S. K. Bogner, R. J. Furnstahl, and A. Schwenk. From low-momentum interactions to nuclear structure. *Prog. Part. Nucl. Phys.*, 65:94–147, 2010.

[S14] S. Srinivas and S. Ramanan. Triplet Pairing in pure neutron matter. *Phys. Rev.*, C94(6):064303, 2016.

[S15] C. Chatterjee, M. Haberichter, and M. Nitta. Collective excitations of a quantized vortex in $^3P_2$ superfluids in neutron stars. *Phys. Rev.*, C96(5):055807, 2017.

[S16] T. Mizushima, K. Masuda, and M. Nitta. $^3P_2$ superfluids are topological. *Phys. Rev.*, B95(14):140503, 2017.

[S17] S. Yasui, C. Chatterjee, and M. Nitta. Symmetry and topology of the boundary of neutron $^3P_2$ superfluids in neutron stars: boojums as surface topological defects. *Phys. Rev.*, C101(2):025204, 2020.

[S18] S. Yasui and M. Nitta. Domain walls in neutron $^3P_2$ superfluids in neutron stars. *Phys. Rev.*, C101(1):015207, 2020.

[S19] T. Mizushima, S. Yasui, and M. Nitta. Critical endpoint and universality class of neutron $^3P_2$ superfluids in neutron stars. *Phys. Rev. Res.*, 2(1):013194, 2020.

[S20] Shigehiro Yasui, Daisuke Inotani, and Muneto Nitta. Coexistence phase of $^1S_0$ and $^3P_2$ superfluids in neutron stars. *Phys. Rev. C*, 101(5):055806, 2020.

[S21] A. P. Schnyder, S. Ryu, A. Furusaki, and A. W. W. Ludwig. Classification of topological insulators and superconductors in three spatial dimensions. *Phys. Rev. B*, 78:195125, 2008.

[S22] A. Kitaev. Periodic table for topological insulators and superconductors. *AIP Conf. Proc.*, 1134:22, 2009.

[S23] S. Autti, V. V. Dmitriev, J. T. Mäkinen, A. A. Soldatov, G. E. Volovik, A. N. Yudin, V. V. Zavjalov, and V. B. Eltsov. Observation of half-quantum vortices in topological superfluid ^{3}He. *Phys. Rev. Lett.*, 117:255301, Dec 2016.

[S24] N. C. Mermin. *Boojums All the Way Through: Communicating Science in a Prosaic Age.* Cambridge University Press., 1990.

[S25] G. E. Volovik. *The Universe in a Helium Droplet.* International Series of Monographs on Physics. Clarendon Press, 2003.

[S26] R. Blaauwgeers, V. B. Eltsov, G. Eska, A. P. Finne, R. P. Haley, M. Krusius, J. J. Ruohio, L. Skrbek, and G. E. Volovik. Shear flow and kelvin-helmholtz instability in superfluids. *Phys. Rev. Lett.*, 89:155301, 2002.

[S27] D. I. Bradley, S. N. Fisher, A. M. Guénault, R. P. Haley, J. Kopu, H. Martin, G. R. Pickett, J. E. Roberts, and V. Tsepelin. Relic topological defects from brane annihilation simulated in superfluid ^{3}He. *Nature Physics*, 4:46–49, 2008.

[S28] J. M. Carlson, S. A. Langer, and J. P. Sethna. Frustration in Modulated Phases: Ripples and Boojums. *Europhysics Letters*, 5:327–331, 1988.

[S29] K. Kasamatsu, H. Takeuchi, and M. Nitta. D-brane solitons and boojums in field theory and Bose-Einstein condensates. *J. Phys. Condens. Matter*, 25:404213, 2013.

[S30] J. P. Gauntlett, R. Portugues, D. Tong, and P. K. Townsend. D-brane solitons in supersymmetric sigma models. *Phys. Rev.*, D63:085002, 2001.

[S31] M. Shifman and A. Yung. Domain walls and flux tubes in N=2 SQCD: D-brane prototypes. *Phys. Rev.*, D67:125007, 2003.

[S32] Y. Isozumi, M. Nitta, K. Ohashi, and N. Sakai. All exact solutions of a 1/4 Bogomol'nyi-Prasad-Sommerfield equation. *Phys. Rev.*, D71:065018, 2005.

[S33] M. Cipriani, W. Vinci, and M. Nitta. Colorful boojums at the interface of a color superconductor. *Phys. Rev.*, D86:121704, 2012.

[S34] Minoru Eto, Yuji Hirono, Muneto Nitta, and Shigehiro Yasui. Vortices and Other Topological Solitons in Dense Quark Matter. *Prog. Theor. Exp. Phys.*, 2014(1):012D01, 2014.

[S35] C. Chatterjee, M. Nitta, and S. Yasui. Quark-hadron continuity under rotation: Vortex continuity or boojum? *Phys. Rev.*, D99(3):034001, 2019.

[S36] J. L. Song, G. W. Semenoff, and F. Zhou. Quantum fluctuation-induced uniaxial and biaxial spin nematics. *Phys. Rev. Lett.*, 98:160408, 2007.

[S37] S. Uchino, M. Kobayashi, M. Nitta, and M. Ueda. Quasi-Nambu-Goldstone Modes in Bose-Einstein Condensates. *Phys. Rev. Lett.*, 105:230406, 2010.

[S38] M. O. Borgh and J. Ruostekoski. Core Structure and Non-Abelian Reconnection of Defects in a Biaxial Nematic Spin-2 Bose-Einstein Condensate. *Phys. Rev. Lett.*, 117(27):275302, 2016. [Erratum: Phys. Rev. Lett.118,no.12,129901(2017)].

[S39] Y. Kawaguchi and M. Ueda. Spinor Bose-Einstein condensates. *Phys. Rept.*, 520:253–381, 2012.
